# Development and Evaluation of 1′-Acetoxychavicol Acetate (ACA)-Loaded Nanostructured Lipid Carriers for Prostate Cancer Therapy

**DOI:** 10.3390/pharmaceutics13040439

**Published:** 2021-03-24

**Authors:** Bavani Subramaniam, Norhafiza M. Arshad, Sharan Malagobadan, Misni Misran, Shaik Nyamathulla, Kein Seong Mun, Noor Hasima Nagoor

**Affiliations:** 1 Institute of Biological Sciences, Faculty of Science, University of Malaya, Kuala Lumpur 50603, Malaysia; bavani90@siswa.um.edu.my; 2 Centre for Research in Biotechnology for Agriculture (CEBAR), University of Malaya, Kuala Lumpur 50603, Malaysia; norhafiza@um.edu.my (N.M.A.); shn.m@outlook.com (S.M.); 3 Department of Chemistry, Faculty of Science, University of Malaya, Kuala Lumpur 50606, Malaysia; misni@um.edu.my; 4 Department of Pharmaceutical Technology, Faculty of Pharmacy, University of Malaya, Kuala Lumpur 50603, Malaysia; nyamathullask@um.edu.my; 5 Department of Pathology, Faculty of Medicine, University of Malaya, Kuala Lumpur 50603, Malaysia; ksmun@ummc.edu.my

**Keywords:** nanostructured lipid carrier, 1′-acetoxychavicol acetate, AMD3100, targeted delivery, prostate cancer

## Abstract

1′-acetoxychavicol acetate (ACA) extracted from the rhizomes of *Alpinia conchigera* Griff (Zingiberaceae) has been shown to deregulate the NF-ĸB signaling pathway and induce apoptosis-mediated cell death in many cancer types. However, ACA is a hydrophobic ester, with poor solubility in an aqueous medium, limited bioavailability, and nonspecific targeting in vivo. To address these problems, ACA was encapsulated in a nanostructured lipid carrier (NLC) anchored with plerixafor octahydrochloride (AMD3100) to promote targeted delivery towards C-X-C chemokine receptor type 4 (CXCR4)-expressing prostate cancer cells. The NLC was prepared using the melt and high sheer homogenization method, and it exhibited ideal physico-chemical properties, successful encapsulation and modification, and sustained rate of drug release. Furthermore, it demonstrated time-based and improved cellular uptake, and improved cytotoxic and anti-metastatic properties on PC-3 cells in vitro. Additionally, the in vivo animal tumor model revealed significant anti-tumor efficacy and reduction in pro-tumorigenic markers in comparison to the placebo, without affecting the weight and physiological states of the nude mice. Overall, ACA-loaded NLC with AMD3100 surface modification was successfully prepared with evidence of substantial anti-cancer efficacy. These results suggest the potential use of AMD3100-modified NLCs as a targeting carrier for cytotoxic drugs towards CXCR4-expressing cancer cells.

## 1. Introduction

Prostate cancer is the second most common cancer affecting men worldwide with approximately 1.3 million cases reported in 2018 [1]. Conventional chemotherapy using semisynthetic plant-derived drugs, such as docetaxel and cabazitaxel, is still regarded as the standardized method of treatment, as approved by the Food and Drug Administration (FDA) [2,3]. One such chemotherapeutic phytoconstituent discovered in recent years is 1′-acetoxychavicol acetate (ACA), extracted from the rhizomes of sub-tropic ginger species *Alpinia galanga* Wild and *Alpinia conchigera* Griff of the Zingiberaceae family [4,5]. ACA has been found to induce apoptosis-mediated cell death in many cancer cell lines including prostate cancer cells with minimal potency towards normal cells [5,6]. Its mechanism of action is associated with the suppression of IKKα/β activation followed by the eventual inhibition of NF-ĸB activation [7]. Nevertheless, as with other plant-derived products, ACA is a poorly soluble ester, causing significant drug delivery problems in vivo [6]. Additionally, the lack of specificity of ACA and several other anti-cancer drugs leads to high drug dosing and prompt adverse reactions, such as systemic toxicity and immunosuppression in cancer patients [8].

As a potential solution to these problems, upgraded treatment methods to specifically target cancer cells with minimal side effects on normal cells have been the focus of researchers in recent years. Strategies involving nanoparticulate carriers with surface modifiers for the active targeting of cancer cells have accumulated with promising results [9,10,11]. An example of a nanocarrier is the nanostructured lipid carrier (NLC), an improvisation of the solid lipid nanoparticle (SLN), with increased structural imperfections for higher drug load [12]. NLCs are made of lipids that have a high affinity towards hydrophobic compounds, entrapping them, and improving their biological distributions and bioavailability. Furthermore, their high surface-area-to-volume ratio is essentially the factor that enhances the rate of drug dispersion in aqueous environments [13]. In addition, the carrier is composed of natural components that pose the least toxicity in vivo as compared to metal-based or synthetic polymer-based nanoparticles [14,15].

Despite their drug protective ability and sustained release properties, NLCs still lack the ability to actively target cancer cells for efficient drug delivery. Hence, several studies have attempted to conduct surface modification of NLCs to target specific receptors on cancer cells [16,17,18,19]. For example, C-X-C chemokine receptor type 4 (CXCR4) is a receptor protein that is overexpressed on many cancer cell lines, including the PC-3 prostate cancer cell line [20,21]. Prostate cancer cell lines that overexpress CXCR4 tend to migrate to organs with high levels of C-X-C motif chemokine ligand 12 (CXCL12), such as the bone marrow [22,23]. The CXCR4/CXCL12 axis promotes the transcription of genes and proteins responsible for tumor development, angiogenesis, and metastasis [22,23,24].

Therefore, targeting CXCR4 on PC-3 cells with its antagonist, such as plerixafor octahydrochloride (AMD3100), is an added advantage. AMD3100 has been found to suppress the migration and invasion tendency of tumor cells to distant sites [25,26]. AMD3100 was also capable of chemo-sensitizing PC model mice to docetaxel and inhibiting the interaction between PC cells and bone marrow stroma [27]. In addition, AMD3100 is positively charged, while the NLC surface is generally negatively charged, allowing electrostatic interactions to form AMD3100-coated NLCs [26].

In light of the above considerations, our main objective was to develop ACA-loaded NLCs with AMD3100 surface modification for a dual function that includes targeting the CXCR4 chemokine receptor and inducing prostate tumor regression. We present here the results from the physico-chemical characterization of the optimized nanoparticles, in vitro cellular uptake, cytotoxicity, migration and invasion assays, and in vivo evaluations of the NLCs on prostate tumor xenografts in nude mice models.

## 2. Materials and Methods

### 2.1. Materials

D, L-1′-acetoxychavicol acetate (ACA) (98.8%) was purchased from LKT Laboratories, Inc. (St Paul, MN, USA), while AMD3100 octahydrochloride was obtained from MedChemExpress (Monmouth Junction, NJ, USA). Cocoa butter from White Naturals (Cape Coral, FL, USA), isopropyl myristate from Friendemann Schmidt (Diviney Court Parkwood, WA, USA), Span 40 from Sigma Aldrich (Darmstadt, Germany), and Tween 80 from Merck (Darmstadt, Germany) were also procured. Deionized water was dispensed from Barnstead NANOpure^®^ Diamond^TM^ (Thermo Scientific, Waltham, MA, USA). PC-3 human prostate cancer cells and RWPE-1 human prostate normal epithelial cells were obtained from American Type Culture Collection (ATCC, Manassas, VA, USA). All other reagents and materials were of analytical reagent grade.

### 2.2. Preparation of NLCs

To prepare the blank NLC, 140 mg of cocoa butter and 60 mg of isopropyl myristate (lipid phase) were melted at 45 °C using a water bath. Simultaneously, 10 mL of deionized water containing 50 mg of Tween 80 and 50 mg of Span 40 (aqueous phase) were heated to 45 °C. The aqueous phase was added to the lipid phase drop by drop. Then, the mixture was homogenized at 20,000 rpm for 20 min before being stored at 4 °C overnight to allow the compounds to form nanostructured particles. For ACA-loaded NLC (ACA-NLC) and AMD3100-coated-ACA-NLC (AMD-ACA-NLC), a similar preparation method was used, but 15 mg ACA was included into the lipid phase before melting at 45 °C. As for the AMD3100-coated blank NLC (AMD-NLC) and AMD-ACA-NLC, 1 mL of AMD3100 (10 mg/mL) was added to 9 mL of the aqueous phase before heating at 45 °C. All the samples were kept at 4 °C storage as nanosuspensions before analysis.

### 2.3. Characterization of NLCs

#### 2.3.1. Particle Size, Polydispersity Index (PDI), and Zeta Potential

Malvern Zetasizer Nano Series (Malvern Instruments, Malvern, UK) uses the dynamic light scattering (DLS) technique to perform size measurement. DLS measures the Brownian motion of the particles and evaluates their size. As for zeta potential measurement, the electrophoretic mobility of the sample was determined using laser Doppler velocimetry (LDV). Mean z-average, PDI, and zeta potential were obtained by diluting the nanoparticle suspension (1:10) with deionized water before loading the sample into a disposable folded capillary zeta cell (Malvern Instruments, Malvern, UK) for measurements in triplicate. The measurement temperature was set at 25 °C.

#### 2.3.2. Entrapment Efficiency

ACA-NLC and AMD-ACA-NLC suspension were subjected to centrifugation (9000 rpm, 25 min, 25 °C) in Vivaspin 6 (10,000 Da MWCO) (Sartorius, Göttingen, Germany) using a tabletop centrifuge. The obtained precipitate was diluted 1:10 in acetonitrile (Merck, Darmstadt, Germany), filtered through a 0.22 μm pore-sized polyethersulfone (PES) (Sartorius, Göttingen, Germany) membrane filter, and run in the Agilent 1220 LC Infinity High Performance Liquid Chromatography (HPLC) system (Agilent Technologies, Santa Clara, CA, USA). The mobile phase conditions were 80:20 (*v:v*) acetonitrile:water elution at a flow rate of 0.8 mL/min, and the absorbance wavelength was 216 nm. An Agilent Poroshell C18 column (4 µm particle size, 4.6 mm × 250 mm) was used. The entrapment efficiency was measured using the equation below, where *C_P_* refers to the concentration of drug in the precipitate, while *C_I_* refers to the initial concentration of drug added.

(1) $$EE\left(\%\right)=\;\frac{C_{P}}{C_{I}}\;\;\times \;100$$

#### 2.3.3. Coating Efficiency

The coating efficiencies of AMD3100 octahydrochloride around AMD-NLC and AMD-ACA-NLC were evaluated by quantification of AMD3100 using a Cary^®^ 50 UV-Vis spectrophotometer (Varian, Inc., Agilent Technologies, Palo Alto, CA, USA). The NLC suspension was subjected to centrifugation (9000 rpm, 25 min, 25 °C) in Vivaspin 6 (10,000 Da MWCO) (Sartorius, Göttingen, Germany) using a tabletop centrifuge. The resultant supernatant was loaded into two clear-side quartz cuvettes with 1 cm path length to measure the amount of AMD3100 at 223 nm wavelength. The coating efficiency was measured using the equation below, where *I_S_* refers to the concentration of AMD3100 in the supernatant, while *I_I_* refers to the initial concentration of AMD3100 added.

(2) $$CE\left(\%\right)=\;\frac{I_{I}-I_{S}}{I_{I}}\;\times 100$$

#### 2.3.4. Transmission Electron Microscopy (TEM)

To observe the shape and morphology of the NLCs, TEM Libra-120 (Zeiss, Jena, Germany) (accelerating voltage 120 kV) was utilized. One drop of NLC suspension was added to a 400-mesh copper-coated carbon grid, before allowing it to rest for 1 min. Then, a filter paper was used to remove the excess liquid droplet before negative staining the sample with 1% (*w/w*) phosphotungstic acid (PTA) solution. The grid was then allowed to dry in a desiccator for 24 h before TEM examination.

#### 2.3.5. Differential Scanning Calorimetry (DSC)

Samples were freeze-dried for 48 h in a freeze-dryer (Labconco, Kansas City, MO, USA) before analysis. About 5 mg of sample was placed in a hermetic pan (TA Instruments, New Castle, DE, USA) with an empty pan serving as the reference. The heating rate was carried out at 10 °C per minute in the range of −20 to 120 °C under an inert nitrogen atmosphere at a flow rate of 420 mL/min using Tzero™ DSC Q20 (TA Instruments, New Castle, DE, USA). The results were analyzed using TA Universal Analysis Software (TA Instruments, New Castle, DE, USA).

#### 2.3.6. Fourier-Transform Infrared Spectroscopy (FTIR)

FTIR spectral data were obtained by placing a miniscule amount (<1 mg) of freeze-dried cocoa butter; ACA, ACA-NLC, and AMD-ACA-NLC samples; and liquid isopropyl myristate oil on the sample area of the FTIR instrument (Perkin Elmer, Waltham, MA, USA). The scanning range was set to 450–4000 cm^−1^ at a spectral resolution of 4 cm^−1^ before analysis.

#### 2.3.7. In Vitro Drug Release

A dialysis method was used to study the in vitro release of ACA. Into a cellulose ester dialysis membrane (10,000 Da) (Spectrum^TM^ Labs, Fisher Scientific, Hampton, NH, USA), ACA, ACA-NLC, and AMD-ACA-NLC solutions of 1 mg/mL concentrations were inserted. The membrane was then suspended in 10 mL of the receiving solution, phosphate-buffered saline (PBS, 0.01 M, pH 7.4). The sample was incubated in an orbital shaker at 37 °C and 90 rpm for 48 h. At specific time intervals within the 48 h, 1 mL of the PBS solution was taken out and replaced with 1 mL of fresh PBS medium. ACA content in the PBS solution was quantified by diluting in acetonitrile (1:10) and running in HPLC. The assay was conducted in triplicate.

### 2.4. Cell Culture

PC-3 human prostate cancer cells were cultured in Rosewell Park Memorial Institute 1640 (RPMI) (Hyclone, Logan, UT, USA) medium supplemented with 10% (*v/v*) fetal bovine serum (FBS), while RWPE-1 human prostate normal epithelial cells were cultured in the complete Keratinocyte Serum-Free Growth Medium (KSFM) (GIBCO, Thermo Fisher Scientific, Waltham, MA, USA). Once the cells reached 80% confluency, they were sub-cultured by detaching the adherent cells with 0.25% trypsin-0.53 mM EDTA solution for PC-3, while 0.05% trypsin-0.53 mM EDTA solution diluted 1:1 in phosphate-buffered saline was used for RWPE-1. The cells were cultured at 37 °C in an incubator with 5% CO_2_ and 95% humidity level.

### 2.5. Cytotoxicity Assay

3-(4,5-dimethylthiazol-2-yl)-2,5-diphenyltetrazolium bromide (MTT) assay was used to assess the cytotoxicity of ACA and the NLC formulations on PC-3 prostate cancer cells and RWPE-1 normal prostate cells. Into 96-well culture plates, 1×10^4^ cells/well were seeded, and the cells were incubated at 37 °C with 5% CO_2_ for 24 h. After incubation, the cells treated with different doses of ACA standalone, blank NLC, ACA-NLC, AMD-NLC, and AMD-ACA-NLC, were incubated for varying durations to observe the effects of the treatment. Next, 20 µL of (5 mg/mL) MTT (Calbiochem, San Diego, CA, USA) was added to each well, and after 90 min of incubation, all the media were removed completely before 200 µL of dimethyl sulfoxide (DMSO) (Merck, Darmstadt, Germany) was added to each well. The plate was kept on the shaker for 10 min. Finally, the absorbance intensity was measured via a micro-titer plate reader (Tecan Sunrise, Männedorf, Switzerland) at an absorbance wavelength of 570 nm, and a reference wavelength of 650 nm. Cell viability was evaluated using the following equation, with Int_S_ referring to the absorbance intensity of the treated cells and Int_Control_ referring to the absorbance intensity of the untreated cells:

(3) $$Cell\;viability\;\left(\%\right)\;=\;\;\frac{Int_{S}}{Int_{Control}}\;\times 100$$

### 2.6. Cellular Uptake Study

A cellular uptake study was conducted by seeding 1 × 10^5^ PC-3 cells/well in a 6-well plate. After attachment, the cells were treated with Coumarin-6 (C6) (Toronto Research Chemicals, Toronto, ON, Canada) solution, C6-NLC, and AMD-C6-NLC at a C6 concentration of 2 µg/mL. At 2, 4, 8, and 24 h post-treatment, the media were discarded, and the cells were washed three times with PBS before their images were captured under a Nikon Eclipse TS 100 fluorescence microscope (Nikon Instruments, Tokyo, Japan). The captured image was quantified for fluorescence intensity using the ImageJ v1.52p (National Institutes of Health, Bethesda, MD, USA) image processing software.

To investigate the cellular uptake mechanisms of the AMD-C6-NLCs, the PC-3 cells were pre-treated with different endocytic inhibitors, such as 0.03 mg/mL nystatin, 0.1 *w/v*% sodium azide, 0.01 mg/mL chlorpromazine, and 100 µM amiloride, at 37 °C for 30 min. The cells were then treated with C6-NLC (control) and AMD-C6-NLC for 2 h without removing the endocytic inhibitors. After treatment, the media were discarded, and the cells were washed three times with PBS to remove any suspending C6 molecule. The cells were imaged under a Nikon Eclipse TS 100 fluorescence microscope (Nikon Instruments, Tokyo, Japan), and their fluorescence intensity measured using ImageJ software. The percentage of cellular uptake was calculated according to the equation below, where F_I_ refers to the fluorescence intensity of the pre-inhibited cells, while F_C_ refers to the fluorescence intensity of cells without pre-inhibition.

(4) $$Percentage\;of\;uptake\;\left(\%\right)\;=\;\;\frac{F_{I}}{F_{C}}\;\;\times \;100$$

As for cellular uptake mediated by CXCR4 targeting, the assay was conducted by pre-incubating the PC-3 cells with varying concentrations of free AMD3100 (0, 10, 20, 50, and 70 µg/mL) for 30 min, before incubating with C6-NLCs and AMD-C6-NLCs for 2 h. The media were then removed, the cells were washed 3 times with PBS, their images captured under a Nikon Eclipse TS 100 fluorescence microscope (Nikon Instruments, Tokyo Japan), and the fluorescence intensity measured using ImageJ software. The relative fluorescence intensity of C6-NLC- and AMD-C6-NLC-treated cells was determined in comparison with the cells treated without AMD3100 inhibition.

### 2.7. Migration Assay

Wound healing assay was conducted to investigate the effects of the different treatments on PC-3 cell migration. In total, 0.6 million cells/well were seeded in 6-well plates with complete media and incubated at 37 °C in 5% CO_2_ for 24 h to allow attachment of cells into monolayers. The next day, the growth media were replaced with serum-free media supplemented with 1 µg/mL of Mitomycin-C (Calbiochem, San Diego, CA, USA), and incubated for 2 h to inhibit cell proliferation. Then, a sterile pipette tip was used to instill wounds of similar size to the monolayer. The cells were washed with 1× PBS twice to remove the cell debris before treatment with ACA, blank NLC, ACA-NLC, AMD-NLC, and AMD-ACA-NLC in serum-free media for 24 h at 37 °C. The images of the wound area before and after treatment were captured using the Nikon Eclipse TS100 inverted fluorescence microscope (Nikon Instruments, Tokyo, Japan), and the migrated area was measured using TScratch software, Version 1.0 (MathWorks Inc., Natick, MA, USA).

### 2.8. Invasion Assay

Cell invasion evaluation was conducted using the transwell invasion assay. PC-3 cells were serum-starved and incubated at 37 °C for 20 h. On the day of the treatment, 24- well transparent PET membranes with 8 μm pore size inserts were coated with 50 µL of 1.5 mg/mL Matrigel (BD Biosciences, San Jose, CA, USA). After the Matrigel hardened, the serum-starved cells were trypsinized, and 500 µL of the cells in serum-free media was added to the insert at 2 × 10^5^ cells/insert. The cells in each insert were treated with ACA, blank NLC, ACA-NLC, AMD-NLC, and AMD-ACA-NLC. Media with 20% (*v/v*) FBS were added to the receiving well as a chemo-attractant. The cells were incubated at 37 °C. After 48 h, cotton swabs were used to gently remove the cells in the upper insert and rinsed twice with PBS. Then, the invading cells on the underside of the membrane were fixed in 100% methanol for 2 min before staining with 1% (*w/v*) methylene blue (Sigma, Ronkonkoma, NY, USA) for 20 min. The inserts were washed with PBS, air-dried, and imaged using the Nikon Eclipse TS100 inverted microscope (Nikon Instruments, Tokyo, Japan) at 200× magnification. The number of invaded cells in four random fields of each insert was counted and recorded.

### 2.9. In Vivo Anti-Tumor Efficacy of the NLCs

In vivo anti-tumor efficacy of the NLCs was evaluated using murine prostate cancer models. Drug doses were established from previous in vitro and in vivo studies with regard to overall mice body weight. Six-week-old athymic male Nude mice (*Nu/Nu*) strains were used in all in vivo tumor xenografting experiments. Induction of tumor was conducted by subcutaneously injecting 100 μL suspensions of PC-3 cells (1 × 10^7^ cells/mL) suspended in 1× PBS and BD Matrigel Matrix HC, at the right flank using 25-gauge needles. The treatments were diluted in 0.9% (*w/v*) sodium chloride (NaCl) solution and administered intraperitoneally when tumor load reached a 50 mm^3^ threshold or higher. The treatments (*n* = 6) were as follows: (i) placebo (0.9% NaCl solution), (ii) ACA standalone (1.56 mg/kg, dissolved in 2% DMSO), (iii) blank NLC (20.8 mg/kg), (iv) ACA-NLC (1.56 mg/kg ACA equivalent), (v) AMD-NLC (20.8 mg/kg), (vi) and AMD-ACA-NLC (1.56 mg/kg ACA equivalent). The mice were treated twice weekly with a 2- to 3-day interval for 4 treatment weeks. Tumor volume and body weight were measured at the beginning of each week, including before and after treatment. A traceable digital caliper was used to measure tumor volumes using the formulae, (major diameter) × 0.5 (minor diameter)^2^. At the end of the treatment, the mice were euthanized, and their organs and tumors were harvested for post-in vivo analysis. The procedures were conducted with approval from the Faculty of Medicine (FOM) Institutional Animal Care and Use Committee (IACUC), University of Malaya (reference number: 2019-200507/IBS/R/BS).

### 2.10. Immunohistochemistry (IHC) Analysis of Tumor Biopsies

Formalin-fixed paraffin-embedded tumor sections from the animal model were subjected to de-paraffinized rehydration and antigen unmasking. Then, the sections were incubated with antibodies specific for p65 (1:400), CXCR4 (1:400), Ki-67 (1:400), and vascular endothelial growth factor (VEGF) (1:600) (Cell Signalling, Danvers, MA, USA). After overnight incubation at 4 °C, SignalStain^®^ Boost IHC Detection Reagent (Horseradish peroxidase-conjugated mouse or rabbit IgG) (Cell Signalling, Danvers, MA, USA) was added to the sections, followed by 3,3′-diaminobenzidine (DAB) solution. Counter-staining was conducted using hematoxylin, before dehydration and mounting. The slides were imaged, and the resulting IHC staining score was calculated using the IHC Profiler tool in ImageJ v1.52p (National Institutes of Health, Bethesda, MD, USA) image processing software.

## 3. Results and Discussion

### 3.1. AMD3100 Surface-Modified ACA-NLC Was Successfully Formulated and Characterized

Table 1 exhibits the physico-chemical properties of the different NLCs formulated in this study. The NLCs had particle sizes that ranged between 115 and 120 nm, which is favorable, because particles between 50 and 200 nm prevent removal by kidneys or recognition by the immune system [28,29,30]. Moreover, the PDI values ranged between 0.15 and 0.2, implying a lack of polydispersed particles that can cause aggregation [31,32]. As for the zeta potential, values between −11 and −30 mV are reasonable for particle repulsion and the prevention of aggregation [33]. Less negative zeta potential values with the addition of ACA and AMD3100 were observed, confirming the successful encapsulation and modification. In addition, the entrapment efficiency of ACA was more than 90%, while the coating efficiency of AMD3100 was more than 70%. This suggests the high affinity of the NLC towards ACA and AMD3100.

**Figure 1:**
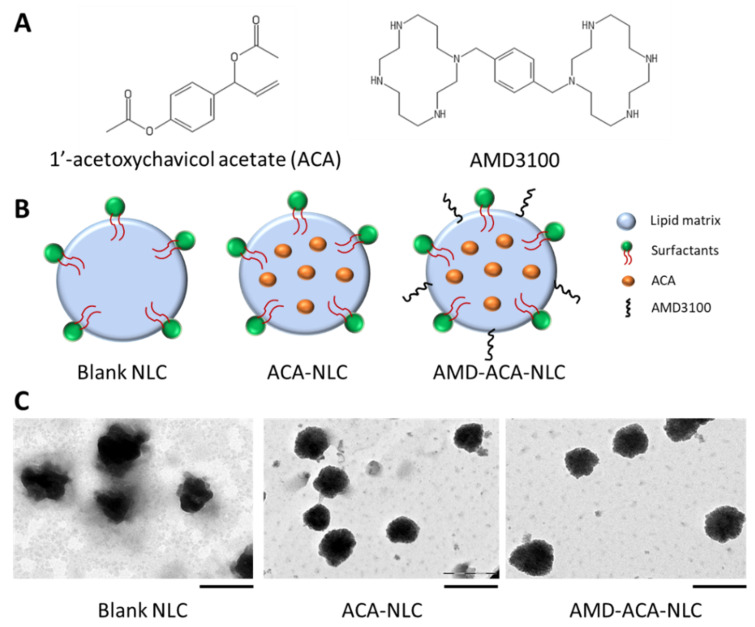
(**A**) Chemical structure of 1′-acetoxychavicol acetate (ACA) and AMD3100. (**B**) Schematic drawings representing blank NLC, ACA-NLC, and AMD-ACA-NLC. (**C**) TEM images of blank NLC, ACA-NLC, and AMD-ACA-NLC. Scale bar = 200 nm.

Under TEM observation (Figure 1), blank NLC, ACA-NLC, and AMD-ACA-NLC had particle size distribution that corresponded to the measured particle size from the DLS analysis. ACA-NLC and AMD-ACA-NLC particles appeared more spherical and regular-shaped than blank NLC, implying an enhancement in particle size distribution and dispersion with the addition of ACA. No visible difference between ACA-NLC and AMD-ACA-NLC could be observed. Overall, the physico-chemical characteristics of blank NLC, ACA-NLC, AMD-NLC, and AMD-ACA-NLC are in accordance with the ideal nanoparticle properties [34].

**Figure 2:**
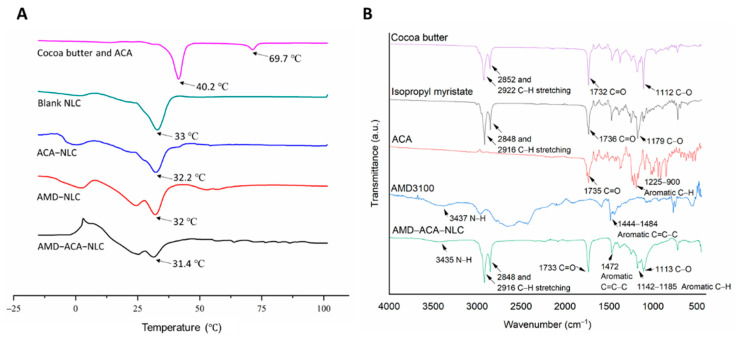
Differential Scanning Calorimetry (DSC) thermograms and Fourier-Transform Infrared Spectroscopy (FTIR) spectrum of samples. (**A**) Thermograms of cocoa butter-ACA physical melt, blank NLC, ACA-NLC, AMD-NLC, and AMD-ACA-NLC. The arrows point to the peak melting temperatures observed in each sample. (**B**) FTIR spectrum of cocoa butter, isopropyl myristate, ACA, AMD3100, and AMD-ACA-NLC. The arrows point to the characteristic peaks of each sample.

To elucidate the thermal properties of the NLCs, Differential Scanning Calorimetry (DSC) analyses were conducted on cocoa butter and ACA physical melt, and the four types of NLCs. Figure 2A shows that the peak melting temperatures of cocoa butter and ACA were 40.2 and 69.7 °C, respectively. The formation of nanosized blank NLCs decreased the melting temperature to 33.0 °C, possibly due to the high surface-area-to-volume ratio [35]. The addition of ACA in ACA-NLC resulted in a slight decrease in melting temperature to 32.2 °C, but the broadened peak displayed an increase in melting enthalpy. ACA-driven internal disorder in the lipid matrix justifies this result [36]. As for AMD-NLC and AMD-ACA-NLC, a broadened double-melting peak was observed at 32 and 31.4 °C respectively, implying semicrystalline polymorph states of the lipid matrix when modified with AMD3100 [37].

FTIR analysis was conducted to determine the functional group characteristics of the NLCs. Figure 2B shows the prominent functional group peaks exhibited by cocoa butter, isopropyl myristate, ACA, AMD3100, and AMD-ACA-NLC. Cocoa butter and isopropyl myristate that make up the lipid matrix displayed similar characteristic peaks at 2848/2850 and 2916/2922 cm^−1^ for C-H stretching of their alkane groups. There were also characteristic peaks at 1732/1736 cm^−1^ for the C=O stretching of their aldehyde groups. The strong peaks at 1112 and 1179 cm^−1^ for cocoa butter and isopropyl myristate, respectively, represent the C-O bonds from the ester group. As for ACA, the characteristic peak at 1735 cm^−1^ represents the C=O of the phenyl ester from its structure. Then, the multiple peaks seen at 900–1225 cm^−1^ revealed the aromatic C-H bends of the benzene. Next, the AMD3100 FTIR analysis resulted in multiple broad peaks. The most noted peaks are the weak N-H peak at around 3437 cm^−1^ and the aromatic C=C-C between 1444 and 1484 cm^−1^. Overall, the AMD-ACA-NLC exhibited similar characteristic peaks to the four compounds. These peaks include the N-H group at around 3435 cm^−1^ from AMD3100, C-H stretching of alkanes from cocoa butter and isopropyl myristate at 2848 and 2916 cm^−1^, C=O and C-O of esters from cocoa butter/isopropyl myristate/ACA at 1733 and 1113 cm^−1^, aromatic C=C-C from AMD3100 at 1472 cm^−1^, and a broadened aromatic C-H peak between 1142 and 1185 cm^−1^ from ACA. The similarity in the functional groups of AMD-ACA-NLC and the raw materials used to formulate it verify the successful formulation of the NLC matrix, encapsulation of ACA, and coating by AMD3100. These findings are in agreement with the FTIR analysis of other nanoformulations [38].

### 3.2. Rate of ACA Release from the NLC Is Sustained

**Figure 3:**
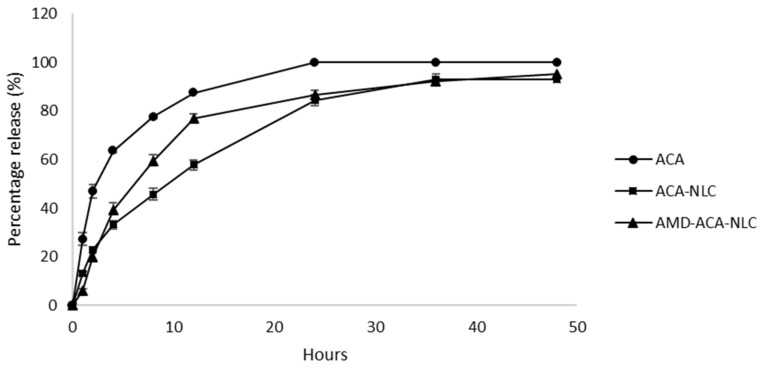
In vitro drug release profile of free ACA, ACA-NLC, and AMD-ACA-NLC solutions over 48 h of incubation at 37 °C. All data are shown as mean ± S.D. of three replicates.

In vitro drug release studies were conducted by quantifying the amount of ACA that permeated through the dialysis membrane to the receiving PBS solution, for 48 h. From Figure 3, it can be observed that the free ACA solution exhibited the fastest rate of release, followed by AMD-ACA-NLC and ACA-NLC. Approximately 50% of ACA was released from free ACA solution, ACA-NLC, and AMD-ACA-NLC within 3, 12, and 8 h of incubation, respectively. Only ACA in free solution could permeate through the membrane at a 100% rate, while ACA-NLC released 93% of ACA within 30 h, and AMD-ACA-NLC released 95% of ACA within 48 h. In comparison to free ACA solution, ACA-NLC and AMD-ACA-NLC showed slower and incomplete release of the drug, similar to other NLC formulations [39,40]. These results reveal that a sustained release of ACA into the external environment occurs when ACA is encapsulated in the NLC matrix with or without AMD3100. This is essential in ensuring that the NLCs reach the targeted region first before releasing their content for their effective therapeutic functions [41,42].

### 3.3. AMD-ACA-NLC Demonstrated Time-Dependent and Enhanced Cellular Uptake via Different Endocytic Pathways

**Figure 4:**
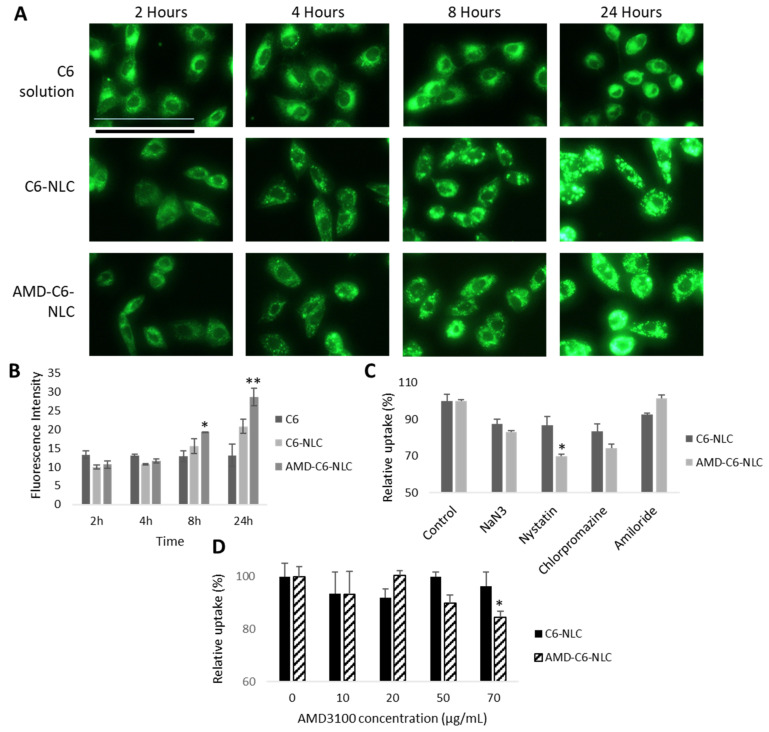
Cellular uptake of C6 solution, C6-NLC and AMD-C6-NLC. (**A**) Representative images of C6 uptake in its free form or when encapsulated in NLC or AMD-NLC after 2, 4, 8, and 24 h of treatment. Scale bar = 50 µm. (**B**) Time-dependent mean fluorescence intensity of cells after different treatments. Statistically significant differences between C6 solution and AMD-C6-NLC are marked by (* *p* < 0.05) and (** *p* < 0.01). All data are shown as mean ± S.D. of three independent replicates. (**C**) Cellular uptake of C6-NLC and AMD-C6-NLC with endocytic inhibitors sodium azide, nystatin, chlorpromazine, and amiloride. (**D**) Cellular uptake of C6-NLC and AMD-C6-NLC with AMD3100 inhibition at different concentrations. Statistically significant differences between C6-NLC and AMD-C6-NLC uptake for C and D are denoted as * *p* < 0.05. All data are shown as mean ± S.D. of three independent replicates.

To study the cellular uptake of blank NLC and AMD-NLC, Coumarin-6 (C6) was loaded into the carrier. C6 is commonly used as a fluorescent dye to imitate hydrophobic drugs or trace particle motility in cell studies [43]. Figure 4A,B show that C6-NLC and AMD-C6-NLC uptake occurred in a time-based manner. The mean fluorescence intensity of C6 solution was higher than C6-NLC and AMD-C6-NLC during the 2nd and 4th hours, but this outcome changed with longer incubation. The uptake of AMD-C6-NLC was significantly higher than C6 solution at the 8th (*p* < 0.05) and 24th hour (*p* < 0.01), while C6-NLC uptake was also higher than C6 solution. This time-dependent uptake of NLCs has been similarly shown in other NLC-based systems from previous studies [38,44]. Nevertheless, the superior uptake of both types of NLC carriers in comparison to the free suspension demonstrates the improved function of the carriers in delivering the active compound [26,45].

To determine the mechanism of uptake for C6-NLC and AMD-C6-NLC, endocytic inhibitors, sodium azide (NaN_3_), nystatin, chlorpromazine, and amiloride were used. As Figure 4C shows, the relative percentages of uptake for C6-NLC and AMD-C6-NLC were reduced under NaN_3_ (cell energy metabolism), nystatin (caveolae-mediated endocytosis), and chlorpromazine (clathrin-mediated endocytosis) pre-treatment, signifying multiple endocytic pathways for the NLC uptake. When C6-NLC and AMD-C6-NLC uptake were compared, a significant difference (*p* < 0.01) was observed under nystatin inhibition. This implied that in the presence of AMD3100, the NLC uptake into PC-3 cells ensued under caveolae-mediated endocytosis. A similar mode of endocytosis was also observed in another AMD3100 modified NLC formulation [26].

Earlier studies have established that CXCR4 is overexpressed in PC-3 cells [22,46]. Hence, the AMD3100 pre-inhibition study was conducted to demonstrate CXCR4-mediated endocytosis of AMD-C6-NLCs into PC-3 cells. Figure 4D shows that a significant reduction in AMD-C6-NLC (*p* < 0.05) uptake was observed under 70 µg/mL of AMD3100 pre-treatment. On the other hand, AMD3100 pre-treatment at all concentrations had no significant effect on C6-NLC uptake. This indicated that AMD3100 was an antagonist of the CXCR4 receptors on PC-3 cells, thus reducing CXCR4-mediated AMD-C6-NLC uptake under excess receptor inhibition [26].

### 3.4. AMD-ACA-NLC Showed Time-Based and Improved Cytotoxicity against PC-3 Cell Lines

**Figure 5:**
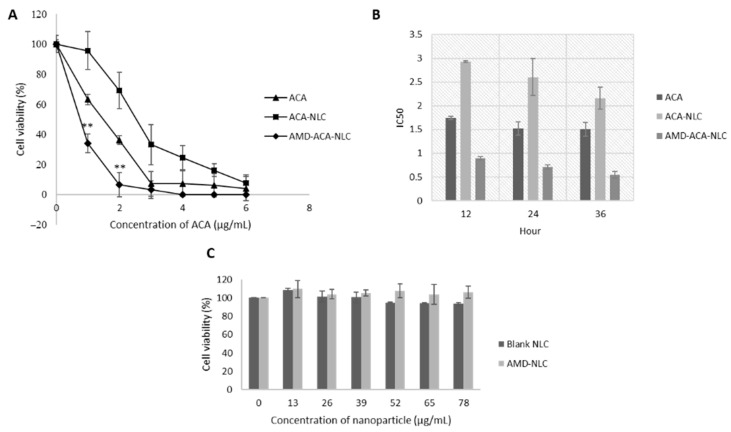
In vitro cytotoxic effects of treatments on PC-3 cell lines. (**A**) Dose-dependent cytotoxicity of ACA, ACA-NLC, and AMD-ACA-NLC on cells after 24 h of incubation. Statistically significant differences between ACA and AMD-ACA-NLC are marked with (** *p* < 0.01). (**B**) Time-dependent IC_50_ of treatments on cells after 12, 24, and 36 h of incubation. (**C**) Dose-dependent cytotoxicity of blank NLC and AMD-NLC on cells after 24 h of incubation. All data are shown as mean ± S.D. of three independent replicates.

Figure 5A shows the dose-dependent cytotoxic effects of ACA, ACA-NLC, and AMD-ACA-NLC on PC-3 cells after 24 h of treatment. The strongest cytotoxic effect was achieved by AMD-ACA-NLC, followed by ACA and then ACA-NLC. The IC_50_ value of AMD-ACA-NLC was 0.71 ± 0.05 µg/mL, which was significantly lower (*p* < 0.01) than ACA’s IC_50_, 1.52 ± 0.14 µg/mL. ACA-NLC had the highest IC_50_ value, which was 2.64 ± 0.39 µg/mL. ACA-NLC’s low cytotoxicity effectiveness can be attributed to a strong drug-carrier hydrophobic bond, thus preventing the complete release of ACA, as with earlier NLC formulations [47,48].

The time-based cytotoxicity of NLCs was prominent in Figure 5B, whereby ACA-NLC’s IC_50_ value of 2.93 ± 0.02 µg/mL at the 12th hour reduced to 2.64 ± 0.39 µg/mL at the 24th hour and 2.16 ± 0.23 µg/mL at the 36th hour. AMD-ACA-NLC also showed a consistent reduction in IC_50_ from 0.89 ± 0.04 µg/mL at the 12th hour, to 0.71 ± 0.05 at the 24th hour and then 0.55 ± 0.07 at the 36th hour. A less noticeable time-based cytotoxicity by ACA standalone caused a decrease in IC_50_ from 1.74 ± 0.04 µg/mL to 1.52 µg/mL between the 12th and 24th hour, which remained unchanged at the 36th hour. Nevertheless, the dose- and time-based cytotoxicity demonstrated that AMD-ACA-NLC elicited the greatest killing effect compared to ACA standalone and ACA-NLC. AMD3100 was found to improve the targeted delivery of ACA to PC-3 cell lines and enhance the latter’s apoptotic activity. Furthermore, Figure 5C shows that neither the blank NLC nor AMD-NLC caused PC-3 cell death after 24 h of treatment. This verified that the cell death is caused by ACA, and AMD3100 potentiates ACA’s toxicity, instead of having any cytotoxic effects of its own. The lack of cytotoxic effects by AMD3100 standalone or NLCs has been demonstrated by several other studies [26,49].

In addition, the cytotoxic evaluation of ACA, ACA-NLC, and AMD-ACA-NLC on the RWPE-1 prostate normal cell line revealed that these treatments exhibited significantly higher IC_50_ values (*p* < 0.01) compared to their respective IC_50_ on PC-3 cells (Table 2). This signifies that ACA and NLC carriers were not harmful to the normal cells as long as the treatment concentration remained below the RWPE-1 cytotoxic concentration.

### 3.5. AMD-ACA-NLC Exhibited Anti-Migration and Anti-Invasion Properties in PC-3 Cells

**Figure 6:**
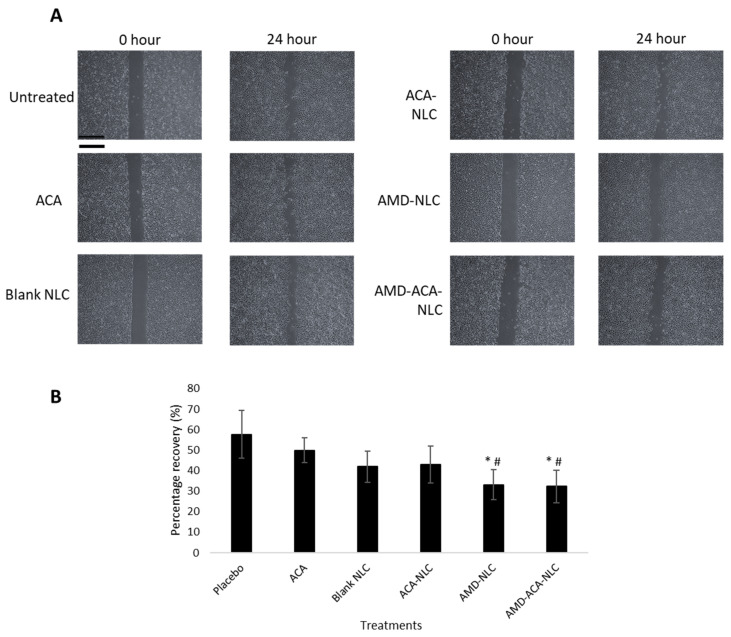
Migration assay conducted on PC-3 cell lines without treatment or with ACA, blank NLC, ACA-NLC, AMD-NLC, and AMD-ACA-NLC treatments. (**A**) Representative images of wound healing assays. Scale bar = 200 µm. (**B**) Percentage recovery of wounds after different treatments. Significant differences in area migrated by cells in comparison to the untreated and ACA-treated cells are marked with * *p* < 0.05 and ^#^ *p* < 0.05, respectively. All data are shown as mean ± S.D. of three independent replicates.

The migration activity of PC-3 cells after treatment with ACA, blank NLC, ACA-NLC, AMD-NLC, and AMD-ACA-NLC was investigated using the wound healing assay. The cells were pre-treated with mitomycin C to inhibit cell proliferation and ensure that the recovery of wound gap is due to cell motility. Figure 6 shows the representative image of wounded cells with different treatments at 0th and 24th hours. The mean percentage of wound recovery for the untreated cells was 57.69 ± 11.7%. As for ACA (49.84 ± 6.1%), blank NLC (41.96 ± 7.6%), and ACA-NLC (42.89 ± 9.0%) treatments, no significant difference compared to the untreated cells was observed. Only AMD-NLC and AMD-ACA-NLC demonstrated significant inhibitions of migration (*p* < 0.05) compared to the untreated and ACA-treated cells, with 33.04 ± 7.3% and 32.24 ± 8.03% recovery, respectively.

**Figure 7:**
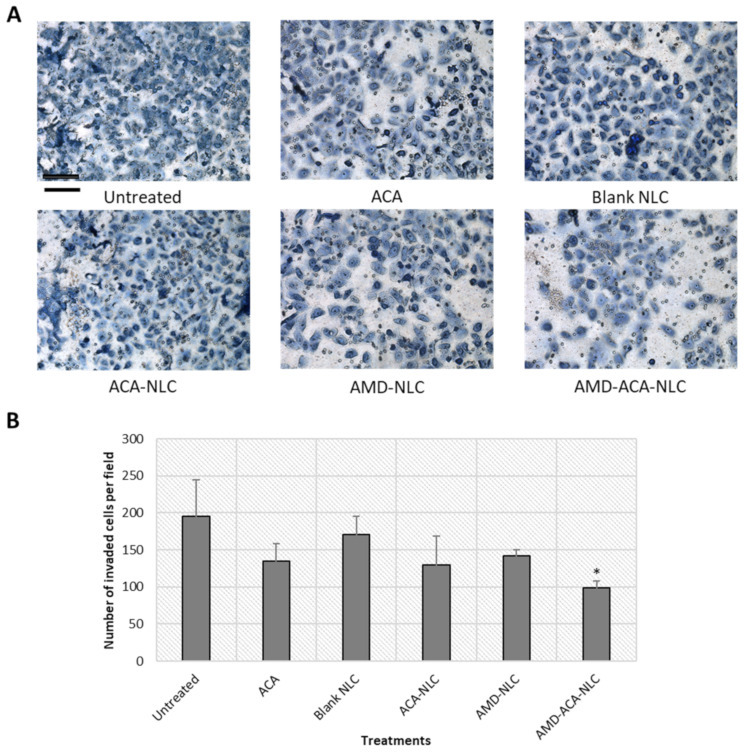
Invasion assay conducted on PC-3 cell lines without treatment or with ACA, blank NLC, ACA-NLC, AMD-NLC, and AMD-ACA-NLC treatments. (**A**) Representative images of transwell inserts. Scale bar = 50 µm. (**B**) Number of invaded cells per field after different treatments. Significant differences in number of invaded cells compared to the untreated are marked with * *p* < 0.05. All data are shown as mean ± S.D. of three independent replicates.

In addition, the transwell invasion assay was conducted to determine the invasion tendency of the cells after treatment. Figure 7 shows that the untreated group exhibited the greatest number of invaded cells per field (195 ± 49) followed by the blank NLC- (171 ± 25), AMD-NLC- (142 ± 8), ACA- (135 ± 23), and ACA-NLC- (129 ± 40) treated groups. A significantly (*p* < 0.05) lower number of invaded cells in comparison to the placebo was demonstrated only by the AMD-ACA-NLC group, with 99 ± 9 cells per field.

All in all, only the AMD-ACA-NLC formulation could inhibit both migration and invasion significantly. Previously, the inhibition of PC-3 cell invasion by ACA has been demonstrated [6]. In this study, however, ACA’s anti-migration and anti-invasion possibilities could only be observed in the presence of AMD3100, as shown by AMD-ACA-NLC. Additionally, AMD-NLC without ACA can also significantly inhibit cell migration. AMD3100 is known to bind to the CXCR4 receptor of cancer cells and prevent the cells from migrating to distant sites expressing CXCL12 ligands [25]. Similarly, several studies have demonstrated the anti-invasive properties of AMD3100 treatment on prostate and breast cancer cell lines [26,46]. Hence, anchoring ACA-NLC with AMD3100 gives the added benefit of preventing the migration and invasion of PC-3 cells.

### 3.6. AMD-ACA-NLC Showed Regression of Tumor Growth

**Figure 8:**
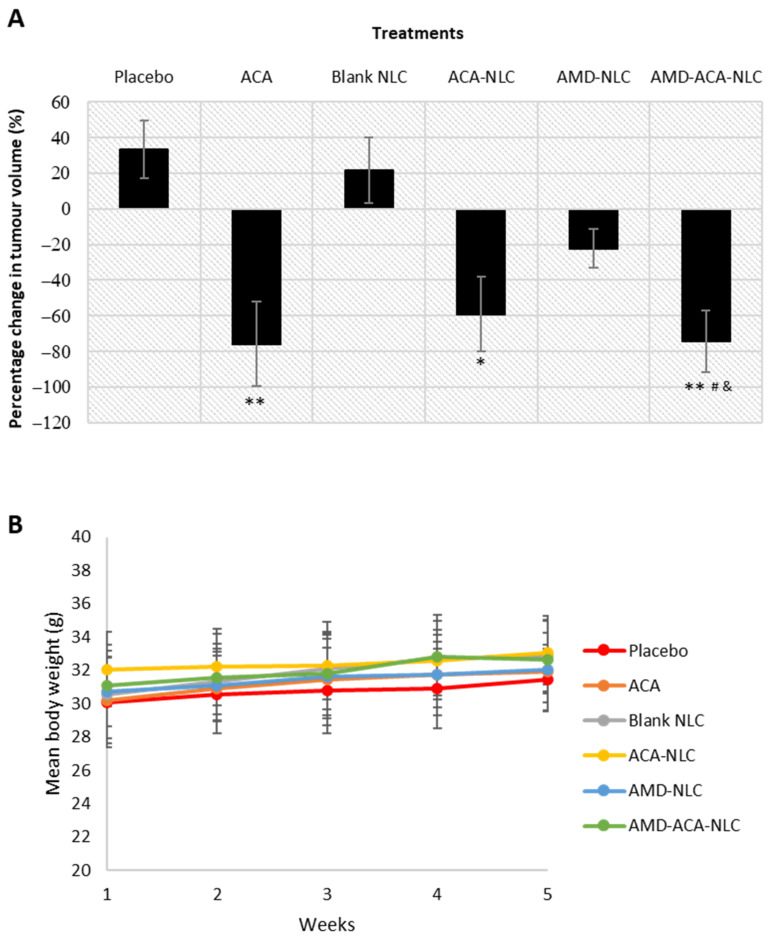
In vivo anti-tumor effects of placebo, ACA, blank NLC, ACA-NLC, AMD-NLC, and AMD-ACA-NLC treatments on *NU*/*NU* mice. (**A**) Percentage change in tumor volume at the end of the treatment. All data are shown as mean value ± SEM of four/five replicates per group. Statistically significant differences from placebo group are shown as * *p* < 0.05 and ** *p* < 0.1, while statistically significant differences from blank NLC and AMD-NLC are shown as ^#^ *p* < 0.05 and ^&^ *p* < 0.05, respectively. (**B**) Mean body weight of PC-3 induced NU/NU mice under various treatments over 28 days. All data are shown as mean value ± SD of five/six replicates per group.

The animal tumor model was established by treating tumor-induced nude mice to saline solution (placebo), ACA, blank NLC, ACA-NLC, AMD-NLC, and AMD-ACA-NLC for 4 weeks. Figure 8A shows the percentage change in tumor growth for all groups at the end of the treatment. The placebo- and blank NLC-treated groups showed an increase in tumor growth, while a significant regression in tumor growth was observed in groups treated with ACA-NLC (*p* < 0.05), ACA (*p* < 0.01), and AMD-ACA-NLC (*p* < 0.01). As for AMD-NLC, the tumor growth declined slightly. Although NLC-based systems are known for their improved drug delivery and enhanced anti-tumor effects [50,51], certain studies have shown inefficient tumor accumulation and a slower rate of drug release by NLC systems compared to the free drug [52,53]. This may explain ACA-NLC’s reduced anti-tumor efficacy as compared to the ACA-free drug, which correlates with its slower rate of in vitro drug release and reduced cytotoxicity. Nevertheless, AMD-ACA-NLC treatment was comparable to the ACA-free drug, implying improved targeted delivery when AMD3100 is used to coat ACA-NLC [26].

Regardless of the lack of anti-tumor superiority by AMD-ACA-NLC and ACA-NLC in comparison to the ACA-free drug, no significant fluctuations in body weight (Figure 8B), behavior, and food/water intake were observed. In addition, the histopathological analysis on major organs, lungs, heart, liver, spleen, and kidneys failed to display any significant inflammation or toxicity induced by the raw materials from the NLC formulations. The results are similar to the placebo-treated group (Figure S1A,B). These verify that the NLC is harmless to the biological environment, and thus can be used in place of hazardous organic solvents required to enhance drug dispersion [54,55].

### 3.7. AMD-ACA-NLC Downregulated Tumor Marker Expressions

**Figure 9:**
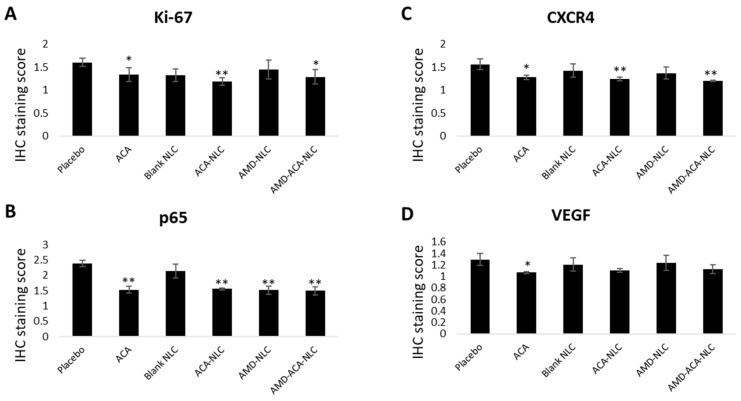
IHC staining score of tumor markers upon treatment with placebo, ACA, blank NLC, ACA-NLC, AMD-NLC, and AMD-ACA-NLC. (**A**) Ki-67, (**B**) CXCR4, (**C**) p65, and (**D**) VEGF protein staining score are shown. Significant difference between treatments and placebo group are indicated as * *p* < 0.05 and ** *p* < 0.01. All data are shown as mean ± S.D. of three independent replicates.

Immunohistochemical (IHC) analyses of tumor biopsies to quantify the cell proliferation marker Ki-67, a chemokine receptor CXCR4, NF-κB regulated gene p65, and inflammatory biomarker VEGF were conducted. Ki-67 is an important antigen in cell proliferation and is used to determine tumor aggressiveness in several types of cancer [56,57]. Figure 9A shows that Ki-67 expression was significantly lower compared to the placebo in ACA-, ACA-NLC-, and AMD-ACA-NLC-treated groups. Previously, a positive correlation between NF-ĸB and Ki-67 levels has been demonstrated [58]; hence, the Ki-67 reduction from ACA, which induced NF-ĸB inactivation in the treatments containing ACA, is as expected.

Next, CXCR4 is a chemokine receptor that promotes tumor growth and mediates cancer cell homing to CXCL-12 producing organs [59,60]. Figure 9B shows that a significant decrease in CXCR4 production was observed in ACA-, ACA-NLC-, and AMD-ACA-NLC-treated groups. It has been previously shown that inactivation of the NF-ĸB pathway causes CXCR4 downregulation [61,62]. Hence, the ACA-induced NF-ĸB pathway inactivation and AMD3100-induced blocking of the CXCR4/CXCL12 axis verify this result [7,46,62].

Moreover, ACA’s association with NF-ĸB inactivation is expected to have direct outcomes on the p65, pro-tumorigenic gene [63]. Figure 9C shows that p65 expression is significantly downregulated in ACA-, ACA-NLC-, AMD-NLC-, and AMD-ACA-NLC-treated groups, in comparison to the placebo. Apart from ACA, AMD3100’s role in blocking the CXCR4/CXCL12 axis to prevent the nuclear translocation of p65 has been demonstrated in several studies [61,64], justifying its role in p65 downregulation.

As for the vascular endothelial growth factor (VEGF), which plays an important role in angiogenesis [65], its expression is only significantly downregulated in the ACA-treated group (Figure 9D). A positive correlation between NF-ĸB and VEGF expression from previous studies [66,67,68] showed that ACA’s inactivation of the NF-ĸB pathway reduced VEGF expressions. Similar results were also observed on A549 and PC-3 tumors treated with ACA [6]. Overall, no significant differences between AMD-ACA-NLC treatment and free ACA could be observed in any of the protein expressions. Previous studies have shown such results where the efficacy of the NLC system in reducing certain pro-tumorigenic protein was similar, and not better than the free drug [69].

## 4. Conclusions

In this study, a nanostructured lipid carrier (NLC) to encapsulate 1′-acetoxychavicol acetate (ACA) for improved dispersion and targeted delivery to CXCR4-expressing prostate cancer cells was formulated. The NLC system exhibited ideal nanoparticle properties and was able to demonstrate the successful encapsulation of ACA and surface modification by the CXCR4 antagonist, AMD3100. The carrier permitted sustained release of ACA, leading to time-based and enhanced cellular uptake and cytotoxicity on PC-3 prostate cancer cells, with minimal cytotoxicity on RWPE-1 normal cells. In addition, AMD3100-modified NLCs significantly prevented the migration and invasion of PC-3 cells. In general, the superior effectiveness of AMD-ACA-NLC in comparison to free ACA was observable in the cell culture studies.

In vivo studies on nude mice models showed that both ACA-NLC and AMD-ACA-NLC significantly regressed tumor growth without negatively affecting the organs or physiological states of the animals, confirming the safety of the raw materials used to design the NLC. In terms of efficacy, AMD-ACA-NLC treatment was significantly effective in comparison to the placebo but not the free ACA. Additionally, the immunohistochemical evaluation of tumor biopsies showed a favorable reduction in tumorigenic proteins under the ACA-NLC and AMD-ACA-NLC treatments. Although an enhanced in vivo efficacy of AMD-ACA-NLC could not be demonstrated compared to free ACA, future studies can be conducted to study the pharmacokinetic profile of the drug-carrier system to optimize the use of the NLC and design therapeutic doses that show significant effectiveness against the free drug in an animal model. Nevertheless, the NLC system was able to exhibit substantial anti-cancer properties in vitro and in vivo on prostate cancer cells and has high potential to be studied in other cancer types in future.

## 5. Patent

This work was patented as Nagoor, N.H., Misran, M., Arshad, N.M., and Subramaniam, B. (2020). Cancer cell-targeted drug composition. Malaysian patent application no: PI2020005770. Filing date: 5 November 2020.

## Figures and Tables

**Table 1:** Physico-chemical properties of the nanostructured lipid carriers (NLCs). All data are expressed as mean ± SD of three replicates.

Samples	Particle Size (nm)	PDI	Zeta Potential (mV)	Entrapment Efficiency (ACA)	Coating Efficiency (AMD3100)
Blank NLC	116.9 ± 0.60	0.204 ± 0.01	−30.9 ± 1.41	-	-
ACA-NLC	115.0 ± 1.27	0.185 ± 0.01	−23.1 ± 0.78	94.0 ± 3.77	-
AMD-NLC	115.2 ± 0.36	0.145 ± 0.02	−12.0 ± 0.94	-	77.3 ± 3.21
AMD-ACA-NLC	120.7 ± 2.07	0.145 ± 0.02	−11.9 ± 0.27	91.3 ± 2.54	74.9 ± 5.17

**Table 2:** IC_50_ values of ACA, ACA-NLC, and AMD-ACA-NLC treatments on RWPE-1 cells. All data are expressed as mean ± SD of three independent replicates.

Cell Line	Treatments	IC_50_ (µg/mL)	
		12 h	24 h
RWPE-1	ACA	>6	>6
	ACA-NLC	4.81 ± 0.11	4.47 ± 0.66
	AMD-ACA-NLC	4.96 ± 0.01	5.12 ± 0.50

## Data Availability

The data presented in this study are available on request from the corresponding author.

## Supplementary Materials

The following are available online at https://www.mdpi.com/1999-4923/13/4/439/s1, Figure S1: (A) Toxicity evaluations in the liver, heart, lungs, spleens, and kidneys of PC-3 bearing NU/NU mice, 28 days post-treatment with different treatments regimens. (B) Hematoxylin and eosin staining were conducted on paraffin-embedded tissue sections. The magnifications are stated below each image.

## References

[1] International Agency for Research on Cancer (2020). Global Cancer Observatory [Homepage on the Internet]. https://gco.iarc.fr.

[2] Picus J., Schultz M. (1999). Docetaxel (Taxotere) as monotherapy in the treatment of hormone-refractory prostate cancer: Preliminary results. Semin. Oncol..

[3] Antonarakis E.S., Paller C.J. (2011). Cabazitaxel: A novel second-line treatment for metastatic castration-resistant prostate cancer. Drug Des. Dev. Ther..

[4] Kondo A., Ohigashi H., Murakami A., Suratwadee J., Koshimizu K. (1993). 1′-acetoxychavicol acetate as a potent inhibitor of tumor promoter-induced Epstein-Barr Virus activation from Languas galanga, a traditional Thai condiment. Biosci. Biotechnol. Biochem..

[5] Awang K., Azmi M.N., Aun L.I., Aziz A.N., Ibrahim H., Nagoor N.H. (2010). The Apoptotic Effect of 1′S-1′-Acetoxychavicol Acetate from Alpinia Conchigera on Human Cancer Cells. Molecules.

[6] Arshad N.M., In L.L., Tchen Lin Soh M.N., Ibrahim H., Awang K., Dudich E., Tatulov E., Nagoor N.H. (2015). Recombinant human alpha fetoprotein synergistically potentiates the anti-cancer effects of 1′-S-1′-acetoxychavicol acetate when used as a complex against human tumours harbouring AFP-receptors. Oncotarget.

[7] La L.L., Arshad N.M., Ibrahim H., Azmi M.N., Awang K., Nagoor N.H. (2012). 1′-Acetoxychavicol acetate inhibits growth of human oral carcinoma xenograft in mice and potentiates cisplatin effect via proinflammatory microenvironment alterations. BMC Complement. Altern. Med..

[8] Blanco E., Hsiao A., Ruiz-Esparza G.U., Landry M.G., Meric-Bernstam F., Ferrari M. (2011). Molecular-targeted nanotherapies in cancer: Enabling treatment specificity. Mol. Oncol..

[9] Zhang Y., Huang Y., Li S. (2014). Polymeric Micelles: Nanocarriers for Cancer-Targeted Drug Delivery. AAPS PharmSciTech.

[10] Torchilin V.P. (2007). Targeted pharmaceutical nanocarriers for cancer therapy and imaging. AAPS J..

[11] Kumari P., Ghosh B., Biswas S. (2016). Nanocarriers for cancer-targeted drug delivery. J. Drug Target..

[12] Müller R., Radtke M., Wissing S. (2002). Nanostructured lipid matrices for improved microencapsulation of drugs. Int. J. Pharm..

[13] Mishra B., Patel B.B., Tiwari S. (2010). Colloidal nanocarriers: A review on formulation technology, types and applications toward targeted drug delivery. Nanomed. Nanotechnol. Biol. Med..

[14] Faraji A.H., Wipf P. (2009). Nanoparticles in cellular drug delivery. Bioorg. Med. Chem..

[15] Sundar D.S., Antoniraj M.G., Kumar C.S., Mohapatra S.S., Houreld N.N., Ruckmani K. (2016). Recent Trends of Biocompatible and Biodegradable Nanoparticles in Drug Delivery: A Review. Curr. Med. Chem..

[16] Sun M., Zhu Z., Wang H., Jin S., Yang X., Han C., Pan W. (2017). Polyarginine and PEG-AEYLR comodified nanostructured lipid carrier: 10 mol% uncleavable PEG-AEYLR showed no shielding effect to polyarginine in vitro while maintaining good tumor targeting in vivo. Artif. Cells Nanomed. Biotechnol..

[17] Wang H., Sun M., Li N., Yang X., Han C., Pan W. (2017). Redox sensitive PEG controlled octaarginine and targeting peptide co-modified nanostructured lipid carriers for enhanced tumour penetrating and targeting in vitro and in vivo. Artif. Cells Nanomed. Biotechnol..

[18] Kong F., Han Y., Zhang Y., Li D., Chen Y., Sun J. (2014). Transferrin-modified nanostructured lipid carriers as multifunctional nanomedicine for codelivery of DNA and doxorubicin. Int. J. Nanomed..

[19] Huang R., Li J., Kebebe D., Wu Y., Zhang B., Liu Z. (2018). Cell penetrating peptides functionalized gambogic acid-nanostructured lipid carrier for cancer treatment. Drug Deliv..

[20] Liles W.C., Broxmeyer H.E., Rodger E., Wood B., Hübel K., Cooper S., Hangoc G., Bridger G.J., Henson G.W., Calandra G. (2003). Mobilization of hematopoietic progenitor cells in healthy volunteers by AMD3100, a CXCR4 antagonist. Blood.

[21] Sun Y.-X., Wang J., Shelburne C.E., Lopatin D.E., Chinnaiyan A.M., Rubin M.A., Pienta K.J., Taichman R.S. (2003). Expression of CXCR4 and CXCL12 (SDF-1) in human prostate cancers (PCa) in vivo. J. Cell. Biochem..

[22] Taichman R.S., Cooper C., Keller E.T., Pienta K.J., Taichman N.S., McCauley L.K. (2002). Use of the stromal cell-derived factor-1/CXCR4 pathway in prostate cancer metastasis to bone. Cancer Res..

[23] Aiuti A., Webb I., Bleul C., Springer T., Gutierrez-Ramos J. (1997). The Chemokine SDF-1 Is a Chemoattractant for Human CD34+ Hematopoietic Progenitor Cells and Provides a New Mechanism to Explain the Mobilization of CD34+ Progenitors to Peripheral Blood. J. Exp. Med..

[24] Chatterjee S., Azad B.B., Nimmagadda S. (2014). The Intricate Role of CXCR4 in Cancer. Adv. Cancer Res..

[25] Slettenaar V.I., Wilson J.L. (2006). The chemokine network: A target in cancer biology?. Adv. Drug Deliv. Rev..

[26] Li H., Wang K., Yang X., Zhou Y., Ping Q., Oupicky D., Sun M. (2017). Dual-function nanostructured lipid carriers to deliver IR780 for breast cancer treatment: Anti-metastatic and photothermal anti-tumor therapy. Acta Biomater..

[27] Domanska U.M., Timmer-Bosscha H., Nagengast W.B., Oude Munnink T.H., Kruizinga R.C., Ananias H.J., Kliphuis N.M., Huls G., De Vries E.G., de Jong I.J. (2012). CXCR4 Inhibition with AMD3100 Sensitizes Prostate Cancer to Docetaxel Chemotherapy. Neoplasia.

[28] Maeda H., Wu J., Sawa T., Matsumura Y., Hori K. (2000). Tumor vascular permeability and the EPR effect in macromolecular therapeutics: A review. J. Control. Release.

[29] Torchilin V. (2011). Tumor delivery of macromolecular drugs based on the EPR effect. Adv. Drug Deliv. Rev..

[30] Li Y., Wang J., Wientjes M.G., Au J.L. (2012). Delivery of nanomedicines to extracellular and intracellular compartments of a solid tumor. Adv. Drug Deliv. Rev..

[31] Zhang J., Fan Y., Smith E. (2009). Experimental Design for the Optimization of Lipid Nanoparticles. J. Pharm. Sci..

[32] Das S., Chaudhury A. (2011). Recent Advances in Lipid Nanoparticle Formulations with Solid Matrix for Oral Drug Delivery. AAPS PharmSciTech.

[33] Mitri K., Shegokar R., Gohla S., Anselmi C., Müller R.H. (2011). Lipid nanocarriers for dermal delivery of lutein: Preparation, characterization, stability and performance. Int. J. Pharm..

[34] Subramaniam B., Siddik Z.H., Nagoor N.H. (2020). Optimization of nanostructured lipid carriers: Understanding the types, designs, and parameters in the process of formulations. J. Nanopart. Res..

[35] Jenning V., Thünemann A.F., Gohla S.H. (2000). Characterisation of a novel solid lipid nanoparticle carrier system based on binary mixtures of liquid and solid lipids. Int. J. Pharm..

[36] Pornputtapitak W., Pantakitcharoenkul J., Teeranachaideekul V., Sinthiptharakoon K., Sapcharoenkun C., Meemuk B. (2019). Effect of Oil Content on Physiochemical Characteristics of γ-Oryzanol-Loaded Nanostructured Lipid Carriers. J. Oleo Sci..

[37] Makoni P.A., Kasongo K.W., Walker R.B. (2019). Short Term Stability Testing of Efavirenz-Loaded Solid Lipid Nanoparticle (SLN) and Nanostructured Lipid Carrier (NLC) Dispersions. Pharmaceutics.

[38] Pillai S.C., Borah A., Jindal A., Jacob E.M., Yamamoto Y., Kumar D.S. (2020). BioPerine Encapsulated Nanoformulation for Overcoming Drug-Resistant Breast Cancers. Asian J. Pharm. Sci..

[39] Ghate V.M., Lewis S.A., Prabhu P., Dubey A., Patel N. (2016). Nanostructured lipid carriers for the topical delivery of tretinoin. Eur. J. Pharm. Biopharm..

[40] Tiwari R., Pathak K. (2011). Nanostructured lipid carrier versus solid lipid nanoparticles of simvastatin: Comparative analysis of characteristics, pharmacokinetics and tissue uptake. Int. J. Pharm..

[41] Dash S., Murthy P.N., Nath L., Chowdhury P. (2010). Kinetic modeling on drug release from controlled drug delivery systems. Acta Pol. Pharm. Drug Res..

[42] Perrie Y., Rades T. (2012). FASTtrack Pharmaceutics: Drug Delivery and Targeting.

[43] Finke J.H., Richter C., Gothsch T., Kwade A., Büttgenbach S., Müller-Goymann C.C. (2014). Coumarin 6 as a fluorescent model drug: How to identify properties of lipid colloidal drug delivery systems via fluorescence spectroscopy?. Eur. J. Lipid Sci. Technol..

[44] Xu P., Yin Q., Shen J., Chen L., Yu H., Zhang Z., Li Y. (2013). Synergistic inhibition of breast cancer metastasis by silibinin-loaded lipid nanoparticles containing TPGS. Int. J. Pharm..

[45] Aleanizy F.S., Alqahtani F.Y., Setó S., Al Khalil N., AlEshaiwi L., Alghamdi M., AlQuadeib B., Alkahtani H., Aldarwesh A., Alqahtani Q.H. (2020). Trastuzumab Targeted Neratinib Loaded Poly-Amidoamine Dendrimer Nanocapsules for Breast Cancer Therapy. Int. J. Nanomed..

[46] Zhu W.B., Zhao Z.F., Zhou X. (2019). AMD3100 inhibits epithelial-mesenchymal transition, cell invasion, and metastasis in the liver and the lung through blocking the SDF-1α/CXCR4 signaling pathway in prostate cancer. J. Cell. Physiol..

[47] Emami J., Rezazadeh M., Varshosaz J., Tabbakhian M., Aslani A. (2012). Formulation of LDL Targeted Nanostructured Lipid Carriers Loaded with Paclitaxel: A Detailed Study of Preparation, Freeze Drying Condition, andIn VitroCytotoxicity. J. Nanomater..

[48] Manivasagan P., Bharathiraja S., Bui N.Q., Lim I.G., Oh J. (2016). Paclitaxel-loaded chitosan oligosaccharide-stabilized gold nanoparticles as novel agents for drug delivery and photoacoustic imaging of cancer cells. Int. J. Pharm..

[49] Cho K.S., Yoon S.J., Lee J.Y., Cho N.H., Choi Y.D., Song Y.S., Hong S.J. (2013). Inhibition of tumor growth and histopathological changes following treatment with a chemokine receptor CXCR4 antagonist in a prostate cancer xenograft model. Oncol. Lett..

[50] Nordin N., Yeap S.K., Rahman H.S., Zamberi N.R., Abu N., Mohamad N.E., How C.W., Masarudin M.J., Abdullah R., Alitheen N.B. (2019). In vitro cytotoxicity and anticancer effects of citral nanostructured lipid carrier on MDA MBA-231 human breast cancer cells. Sci. Rep..

[51] Li M., Pei J., Ma Z., Fu J., Chen F., Du S. (2020). Docetaxel-loaded ultrasmall nanostructured lipid carriers for cancer therapy: In vitro and in vivo evaluation. Cancer Chemother. Pharmacol..

[52] Li W., Fu J., Ding Y., Liu D., Jia N., Chen D., Hu H. (2019). Low density lipoprotein-inspired nanostructured lipid nanoparticles containing pro-doxorubicin to enhance tumor-targeted therapeutic efficiency. Acta Biomater..

[53] Stewart M.P., Sharei A.R., Ding X.S., Sahay G., Langer R.S., Jensen K.F. (2016). In vitro and ex vivo strategies for intracellular delivery. Nature.

[54] Rahman H.S., Rasedee A., Othman H.H., Chartrand M.S., Namvar F., Yeap S.K., Samad N.A., Andas R.J., Nadzri N.M., Anasamy T. (2014). Acute Toxicity Study of Zerumbone-Loaded Nanostructured Lipid Carrier on BALB/c Mice Model. BioMed Res. Int..

[55] Nordin N., Yeap S.K., Zamberi N.R., Abu N., Mohamad N.E., Rahman H.S., How C.W., Masarudin M.J., Abdullah R., Alitheen N.B. (2018). Characterization and toxicity of citral incorporated with nanostructured lipid carrier. PeerJ.

[56] Brown D., Gatter K. (2002). Ki67 protein: The immaculate deception?. Histopathology.

[57] Li L.T., Jiang G., Chen Q., Zheng J.N. (2015). Ki67 is a promising molecular target in the diagnosis of cancer (Review). Mol. Med. Rep..

[58] Meteoglu I., Erdoğdu I.H., Tuncyurek P., Coşkun A., Culhaci N., Erkus M., Barutca S. (2015). Nuclear Factor Kappa B, Matrix Metalloproteinase-1, p53, and Ki-67 Expressions in the Primary Tumors and the Lymph Node Metastases of Colorectal Cancer Cases. Gastroenterol. Res. Pract..

[59] Singh S., Singh U.P., Grizzle W.E., Lillard J.W. (2004). CXCL12-CXCR4 interactions modulate prostate cancer cell migration, metalloproteinase expression and invasion. Lab. Investig..

[60] Sun X., Cheng G., Hao M., Zheng J., Zhou X., Zhang J., Taichman R.S., Pienta K.J., Wang J. (2010). CXCL12/CXCR4/CXCR7 chemokine axis and cancer progression. Cancer Metastasis Rev..

[61] Zhi Y., Lu H., Duan Y., Sun W., Guan G., Dong Q., Yang C. (2015). Involvement of the nuclear factor-κB signaling pathway in the regulation of CXC chemokine receptor-4 expression in neuroblastoma cells induced by tumor necrosis factor-α. Int. J. Mol. Med..

[62] Helbig G., Christopherson K.W., Bhat-Nakshatri P., Kumar S., Kishimoto H., Miller K.D., Broxmeyer H.E., Nakshatri H. (2003). NF-kappaB promotes breast cancer cell migration and metastasis by inducing the expression of the chemokine receptor CXCR4. J. Biol. Chem..

[63] Xia Y., Shen S., Verma I.M. (2014). NF-κB, an Active Player in Human Cancers. Cancer Immunol. Res..

[64] Liu Z., Ma C., Shen J., Wang D., Hao J., Hu Z. (2016). SDF-1/CXCR4 axis induces apoptosis of human degenerative nucleus pulposus cells via the NF-κB pathway. Mol. Med. Rep..

[65] Verheul H.M., Pinedo H.M. (2000). The Role of Vascular Endothelial Growth Factor (VEGF) in Tumor Angiogenesis and Early Clinical Development of VEGFReceptor Kinase Inhibitors. Clin. Breast Cancer.

[66] Xie T.-X., Xia Z., Zhang N., Gong W., Huang S. (2010). Constitutive NF-κB activity regulates the expression of VEGF and IL-8 and tumor angiogenesis of human glioblastoma. Oncol. Rep..

[67] Kiriakidis S., Andreakos E., Monaco C., Foxwell B., Feldmann M., Paleolog E. (2003). VEGF expression in human macrophages is NF-κB-dependent: Studies using adenoviruses expressing the endogenous NF-κB inhibitor IκBα and a kinase-defective form of the IκB kinase 2. J. Cell Sci..

[68] Du Z., Zhang H., Gao D., Wang H., Li Y., Liu G. (2006). Significance of VEGF and NF-kB expression in thyroid carcinoma. Chin. Clin. Oncol..

[69] Nordin N., Yeap S.K., Rahman H.S., Zamberi N.R., Mohamad N.E., Abu N., Masarudin M.J., Abdullah R., Alitheen N.B. (2020). Antitumor and Anti-Metastatic Effects of Citral-Loaded Nanostructured Lipid Carrier in 4T1-Induced Breast Cancer Mouse Model. Molecules.

