# Supplementary material for: Development and Evaluation of 1′-Acetoxychavicol Acetate (ACA)-Loaded Nanostructured Lipid Carriers for Prostate Cancer Therapy
Source: Pharmaceutics. 2021 Mar 24;13(4):439. doi: 10.3390/pharmaceutics13040439 (PMC8063947; doi:10.3390/pharmaceutics13040439)
Supplement: Supplementary file 1 [file pharmaceutics-13-00439-s001.pdf]

pharmaceutics

MDPI

Supplementary Materials: Development and Evaluation of 1′-Acetoxychavicol Acetate (ACA)-Loaded Nanostructured Lipid Carriers for Prostate Cancer Therapy

Bavani Subramaniam, Norhafiza M. Arshad, Sharan Malagobadan, Misni Misran, Shaik Nyamathulla, Kein Seong Mun and Noor Hasima Nagoor

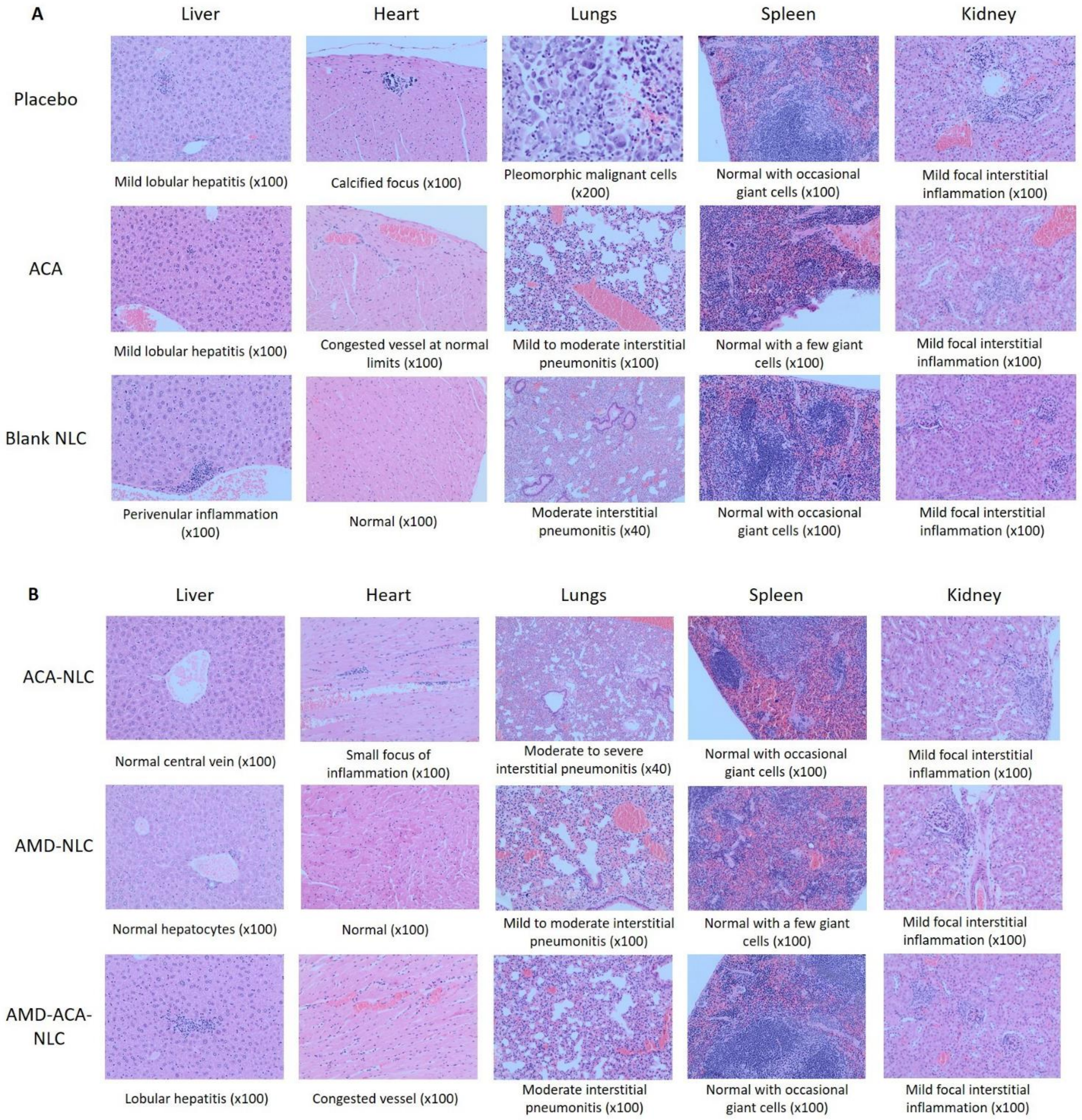

Figure S1. (**A**) Toxicity evaluations in the liver, heart, lungs, spleens, and kidneys of PC-3 bearing NU/NU mice, 28 days post-treatment with different treatments regimens. (**B**) Hematoxylin and eosin staining were conducted on paraf-fin-embedded tissue sections. The magnifications are stated below each image.
